# Molecular Detection of *Rickettsia* *typhi* in Cats and Fleas

**DOI:** 10.1371/journal.pone.0071386

**Published:** 2013-08-06

**Authors:** Maria Mercedes Nogueras, Immaculada Pons, Ana Ortuño, Jaime Miret, Julia Pla, Joaquim Castellà, Ferran Segura

**Affiliations:** 1 Department of Infectious Diseases. Corporació Sanitària i Universitària Parc Taulí - Institut Universitari Parc Taulí – Autonomous University of Barcelona, Sabadell, Barcelona, Spain; 2 Department of Animal Health, Autonomous University of Barcelona, Bellaterra, Barcelona, Spain; 3 Animal Shelter Company (Vallès Oriental), Granollers, Barcelona, Spain; 4 Clinical Veterinary, Sabadell, Barcelona, Spain; 5 Department of Medicine, Universitat Autònoma de Barcelona, Bellaterra, Barcelona, Spain; University of Minnesota, United States of America

## Abstract

**Background:**

*Rickettsia* *typhi* is the etiological agent of murine typhus (MT), a disease transmitted by two cycles: rat-flea-rat, and peridomestic cycle. Murine typhus is often misdiagnosed and underreported. A correct diagnosis is important because MT can cause severe illness and death. Our previous seroprevalence results pointed to presence of human *R* *. typhi* infection in our region; however, no clinical case has been reported. Although cats have been related to MT, no naturally infected cat has been described. The aim of the study is to confirm the existence of *R* *. typhi* in our location analyzing its presence in cats and fleas.

**Methodology/Principal Findings:**

221 cats and 80 fleas were collected from Veterinary clinics, shelters, and the street (2001-2009). Variables surveyed were: date of collection, age, sex, municipality, living place, outdoor activities, demographic area, healthy status, contact with animals, and ectoparasite infestation. IgG against *R* *. typhi* were evaluated by indirect immunofluorescence assay. Molecular detection in cats and fleas was performed by real-time PCR. Cultures were performed in those cats with positive molecular detection. Statistical analysis was carried out using SPSS. A p < 0.05 was considered significant.

Thirty-five (15.8%) cats were seropositive. There were no significant associations among seropositivity and any variables. *R* *. typhi* was detected in 5 blood and 2 cultures. High titres and molecular detection were observed in stray cats and pets, as well as in spring and winter. All fleas were *Ctenocephalides felis*. *R* *. typhi* was detected in 44 fleas (55%), from shelters and pets. Co-infection with *R* *. felis* was observed.

**Conclusions:**

Although no clinical case has been described in this area, the presence of *R* *. typhi* in cats and fleas is demonstrated. Moreover, a considerable percentage of those animals lived in households. To our knowledge, this is the first time *R* *. typhi* is detected in naturally infected cats.

## Introduction

Murine typhus is one of the most prevalent Rickettsioses worldwide distributed, and it is endemic in coastal areas and ports [1]. Its aetiological agent is *Rickettsia* *typhi* , which belongs to typhus group rickettsiae. Two cycles are involved in *R* *. typhi* transmission: a classical cycle rat-flea-rat, and a peridomestic cycle involving cats, dogs, opossums, sheep and their fleas [1].

Murine typhus is often acute and mild [2]. However, it can cause severe illness and death [1,3–6]. Although the lack of a correct diagnosis increases the risk of severity, murine typhus is usually misdiagnosed and underreported and its prevalence is unknown in many countries. Murine typhus may be mistaken for other diseases because of its non-specific symptoms [2–4]. In addition, epidemiological criteria are not always present [3–5].

In Spain, murine typhus has been known for more than 25 years [7,8]. Most clinical cases occur in two regions: *Anda* *luciae* [5,7,8] and the Canary Islands [9–11]. However, sero-epidemiological studies have demonstrated the presence of *R* *. typhi* infection throughout the country [12–15]. For instance, even though no clinical cases have been described in our region, the seroprevalence of *R* *. typhi* infection in human population was 8.8%. Consequently, murine typhus could be misdiagnosis in Spain. For this reason, it is necessary to demonstrate the presence of *R* *. typhi* as well as to detect reservoirs, vectors, and risk factors.

An association between infected cats and murine typhus human cases has been described [4,16]. Our previous results showed that subjects who reported contact with pets tended to have higher *R* *. typhi* seroprevalence [12]. Thus, a peridomestic cycle may be present in our region and cats and fleas could be involved. There are few studies on fleas related to *R* *. typhi* . Moreover, although cats could be susceptible to *R* *. typhi* subclinical infection [16,17], no naturally infected cats have been described up to know. The objectives of the study were to detect *R* *. typhi* in fleas and naturally infected cats, and to demonstrate the presence of *R* *. typhi* in a region where no clinical cases has been reported and, thus, murine typhus may be misdiagnosed.

## Methods

### Geographical area

**Figure 1:**
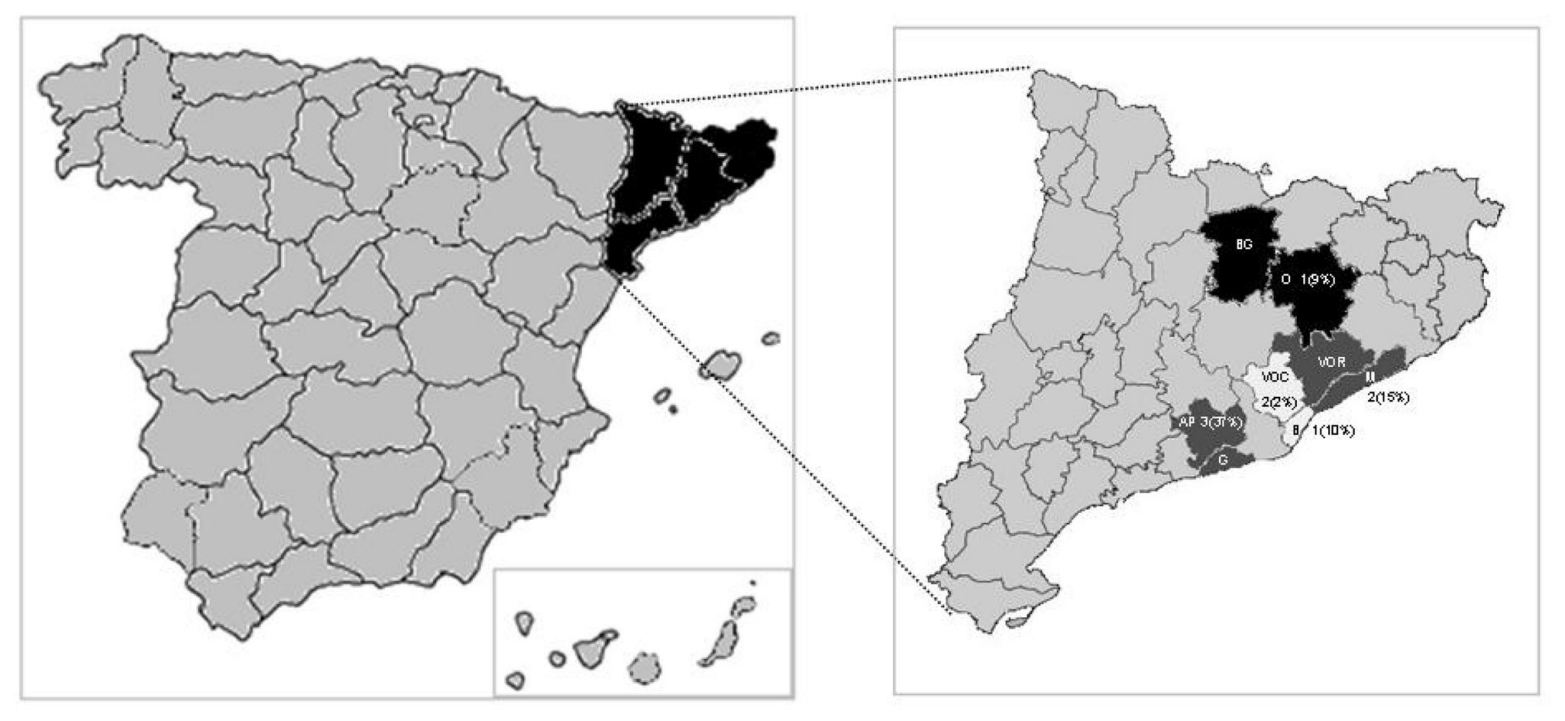
Geographical area of the study. Predominantly urban areas: Barcelonés [B], Baix Llobregat [BL], Vallés Occidental [VOC]. Predominantly suburban areas: Maresme [M], Selva [S], Vallés Oriental [VOR].

The study was undertaken on the central coast of Catalonia, a predominantly urban region in Northeastern Spain. A total of twenty municipalities, which belonged to six areas, participated in the study (Figure 1).

### Samples

Two hundred and twenty-one cats were analysed (January 2001 to March 2009). Forty-four were stray cats subjected to health and reproductive control by municipalities. Their samples were provided by the municipal veterinarians. One hundred and seventy-seven cats were attended at different veterinary clinics. One of these clinics worked together with PROGAT. The latter was a foundation that collected stray cats, controlled their health and left them in the street again. Thus, twenty-four cats attended at veterinary clinics were stray cats.

Variables registered were: date of collection, age, sex, municipality, living place, outdoor activities, demographic area (rural: <5,000 inhabitants; suburban 5,000 to 50,000; urban: > 50,000 inhabitants), healthy status, contact with animals, and ectoparasite infestation.

Blood samples were aseptically collected from the external jugular vein of each cat. One millilitre of blood was introduced in a serum-separating tube. Sera were obtained by centrifugation. Whole-blood samples were collected in sterile EDTA and heparin vacutainers. Samples were frozen at -80^°^ C until used.

Eighty fleas were studied. Sixty-four were collected in kennels of the same areas. Most fleas were collected in the boxes and some were collected on animals. In addition, sixteen fleas were collected on cats attended at veterinary clinics. Fleas were identified according to taxonomic keys. Fleas were immersed in a solution of 70% ethanol, washed in sterile distilled water, dried, and individually transferred to tubes. Samples were stored at 4^°^ C.

### Indirect immunofluorescence assay

Antibodies titres against *Rickettsia* *typhi* were evaluated by indirect immunofluorescence assay (IFA) using a commercial antigen ( *R. typhi* *-R. rickettsii* IgG, Focus Technologies, Inc., Herndon, VA). Briefly, 25µL of twofold dilutions of cat sera in phosphate-buffered saline (PBS) -3% non-fat dry milk were applied to the antigens. Sera of a seropositive cat and a seronegative cat, kindly obtained from Veterinary Faculty, were added to each experiment as positive and negative controls. The slides were incubated in a humidified chamber at 37^°^ C for 30 min. Two washes (10 min) in PBS and one wash (5 min) in water were performed to remove unbound immunoglobulins. Slides were air dried. Binding sera were detected using a fluorescein isothiocyanate-labelled anti-cat IgG (Sigma-Aldrich Química, S.A., Madrid) diluted 1/128 in PBS -0.01% Evans Blue (bioMérieux, S.A., Madrid). The slides were incubated and washed as described above. The slides were examined with a fluorescence microscope at 400x. The highest dilution, at which distinct and specific fluorescence was seen, was scored as the end-point titer for the serum sample. Titres ≥ 1/64 were considered positive. The samples were subjectively evaluated and independently graded by two of the authors.

### Molecular detection

DNA was obtained from cats samples collected in an EDTA whole-blood vacutainer, and from fleas individually triturated in brain-heart infusion (BHI) (bioMérieux, Marcy-l’Etoile, France) [18]. Masterpure DNA purification kit (Epicentre, Madison, Wisconsin) was used according to the manufacturer’s instructions. This kit included a Uracil DNA Glycosylase (UNG) and deoxyuracil triphosphates (dUTP) as well as a Hot start DNA polymerase. DNA from cultures of *Rickettsia* *typhi* (kindly obtained from the Unité de Rickettsies, France) was obtained and used as positive control. Measures to avoid contamination were performed using separate and dedicated rooms for DNA extraction and molecular detection.

DNA samples were tested by *R* *. typhi* -specific real-time PCR targeting the gene for *omp*B [19]. When there was enough DNA, a second real-time PCR assay targeting the gene *glt*A was carried out [20]. PCR assays were performed and analysed using 7500 thermocycler (Applied Biosystems). Two negative controls (DNA-free water as template) and one positive control (purified *R* *. typhi* DNA) were included in all assays. PCRs were set up in a UV-sterilized workstation. Each sample was assayed three times. Amplification products were purified by Exosap-it (GE Healthcare, Bunkinghamshire, UK) and sequenced on 3130 Genetic Analyzer (Applied Biosystems) using a BigDye Terminator v 3.1 Cycle Sequencing Kit (Applied Biosystems, Foster City, CA). The sequences were compared with those in the GenBank by BLAST program (megablast algorithm).

### Culture

Whole-blood samples collected in a heparin vacutainer were used. Three hundred microlitres of blood were added to two shell vials seeded with Vero cells (African green monkey epithelial cells). A shell vial is a tube with a coverslip that contains the cell monolayer. After adding blood, the shell vials were centrifuged at 700*g* for one hour. During centrifugation, microorganisms were in close proximity to the cells and, thus, infection efficiency was enhanced. The shell vials were incubated at 32^°^ C. Every week, the medium was replaced. On day 20, cell monolayer was scraped with glass beads. Part of the culture collected was transferred to a confluent monolayer of Vero cells in a 25 cm^2^ culture flask.

On day 40, monolayers were scraped with glass beads and cultures were collected. A drop was placed on a slide and evaluated by Giménez staining. DNA was obtained from each culture. Presence of *R* *. typhi* DNA was analysed using *omp*B *R* *. typhi* -specific PCR [19] and sequencing as described above.

Two slides were prepared for indirect immunofluorescence assay (IFA). Cultures collected were deposited onto the slides, air dried and fixed with acetone. They were incubated with murine typhus group (MT) positive control or spotted fever group (SFG) positive control of the commercial kit for *R. typhi* *-R. rickettsii* IgG detection (Focus Technologies, Inc., Herndon, VA). These controls contain antibodies against Typhus Fever group and Spotted Fever group rickettsiae, respectively. Three wells of a commercial slide, with antigen to *R. typhi* *-R. rickettsii*, were incubated with MT positive control, SFG positive control, and negative control of the same kit. IFA was performed according to the manufacturer’s instructions of the kit.

### Statistical analysis

To achieve an accuracy of 5.0% in the estimation of a confidence interval using a normal asymptotic finite population correction for the bilateral 95%, assuming that the expected proportion were the highest prevalences found in the literature (worst cases) and that the total size of the populations were 1000, the sample size was calculated.

Data analysis was carried out using the software application SPSS Statistics 18.0. Univariate analysis was performed using Chi-square test, Fisher exact test, and Mann–Whitney U test. A p < 0.05 was considered significant.

### Ethics Statement

This study was approved by the Ethical Committee of Corporació Sanitària i Universitària Parc Taulí. This study was adhered to the Animal Protection Law (5/1995) of the Government of Catalonia, and RD1201/2005 of the Government of Spain, based on European Union directives 86/609/CEE and 2003/65/CE. Animal owners consented to have their cats involved in the study.

## Results

### Cats’ study

#### Study population

The clinical and epidemiological characteristics are shown in Table 1. The mean age was 3.9 ± 4.2 years (0.4-17 years). Most samples were collected between February and July (74.6%). Samples of cats attended at veterinary clinics were collected throughout the year, whereas sample of stray cats controlled by municipalities were collected in May (88.9%) and June (11.1%).

**Table 1:** Demographic information from cats tested for antibodies to *Rickettsia* *typhi* .

**Variables**	**Total N (%^1^**)	**Positive N (%^1^)**
**Sex**		
**Male**	103 (46.6)	19 (54.3)
**Female**	114 (51.6)	16 (45.7)
**Age**		
**Kitten**	60 (27.1)	9 (25.7)
**Adult**	154 (69.7)	26 (74.3)
**Living place**		
**Apartment**	54 (24.4)	12 (34.3)
**House**	94 (42.5)	13 (37.1)
**Stray**	68 (30.8)^2^	10 (28.6)
**Demographic area**		
**Urban**	183 (82.8)	28 (80)
**Suburban**	26 (11.8)	6 (17.1)
**Rural**	12 (5.4)	1 (2.9)
**Regions**		
**Barcelonés**	44 (19.9)	9 (25.7)
**Baix Llobregat**	1 (0.5)	0 (0)
**Maresme**	19 (8.6)	3 (8.6)
**Selva**	1 (0.5)	1 (2.9)
**Vallés Occidental**	146 (66.2)	22 (62.9)
**Vallés Oriental**	10 (4.5)	0 (0)
**On the coast**		
**Yes**	59 (26.7)	11 (31.4)
**No**	162 (73.3)	24 (68.6)
**Contact with animals**		
**Yes**	172 (77.8)	27 (77.1)
**No**	37 (16.7)	8 (22.9)
**Contact with cats**	132 (59.7)	18 (51.4)
**Contact with dogs**	48 (21.7)	9 (25.7)
**Contact with other animals**	5 (2.3)^3^	1 (2.9)^4^
**Illness**		
**Yes**	29 (13.1)^5^	7 (24.1)^6^
**No**	160 (72.4)	22 (75.9)
**TOTAL**	**221**	**35**

^1^ The percentage is calculated against the total number of animals that belong to the study population or the seropositive population. ^2^ 24 (10.9%) stray cats were attended at PROGAT foundation, and 44 (19.9%) belonged to groups of stray cats subjected to control by municipalities (controlled cats). ^3^ Other animals (total population): birds, mice, sheep, wild animals and rabbits; ^4^ Other animals (seropositive population): bird and mice. ^5^ Diseases (total population): abscess, dehydration, abortion, sepsis, cystitis, conjunctivitis, anaemia, diabetes mellitus, diarrhoea, fever, gingivitis, hepatic diseases, respiratory diseases, Feline immunodeficiency, poisoning, feline leukaemia, breast lump, nasal lump, mouth infection, urinary tract infection, worms, and uterus infection. No association was observed between the presence of illness and any of the variables. ^6^ Diseases (seropositive population): abscess, cystitis, mouth infection, hepatic diseases, gingivitis, and respiratory diseases (two cats)

**Figure 2:**
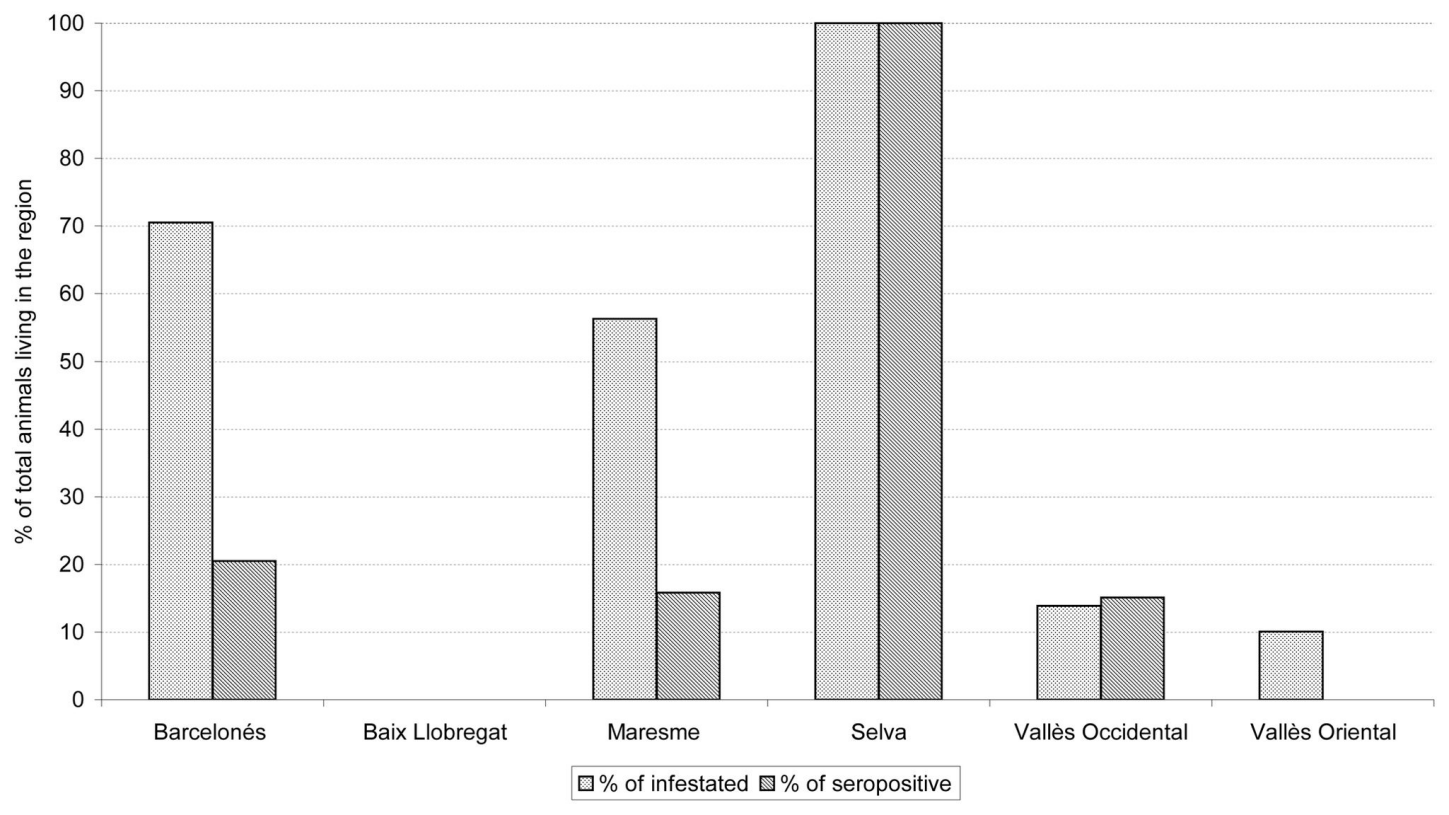
Geographical distribution: percentage of infested and seropositive cats compared to the entire population of each region.

**Figure 3:**
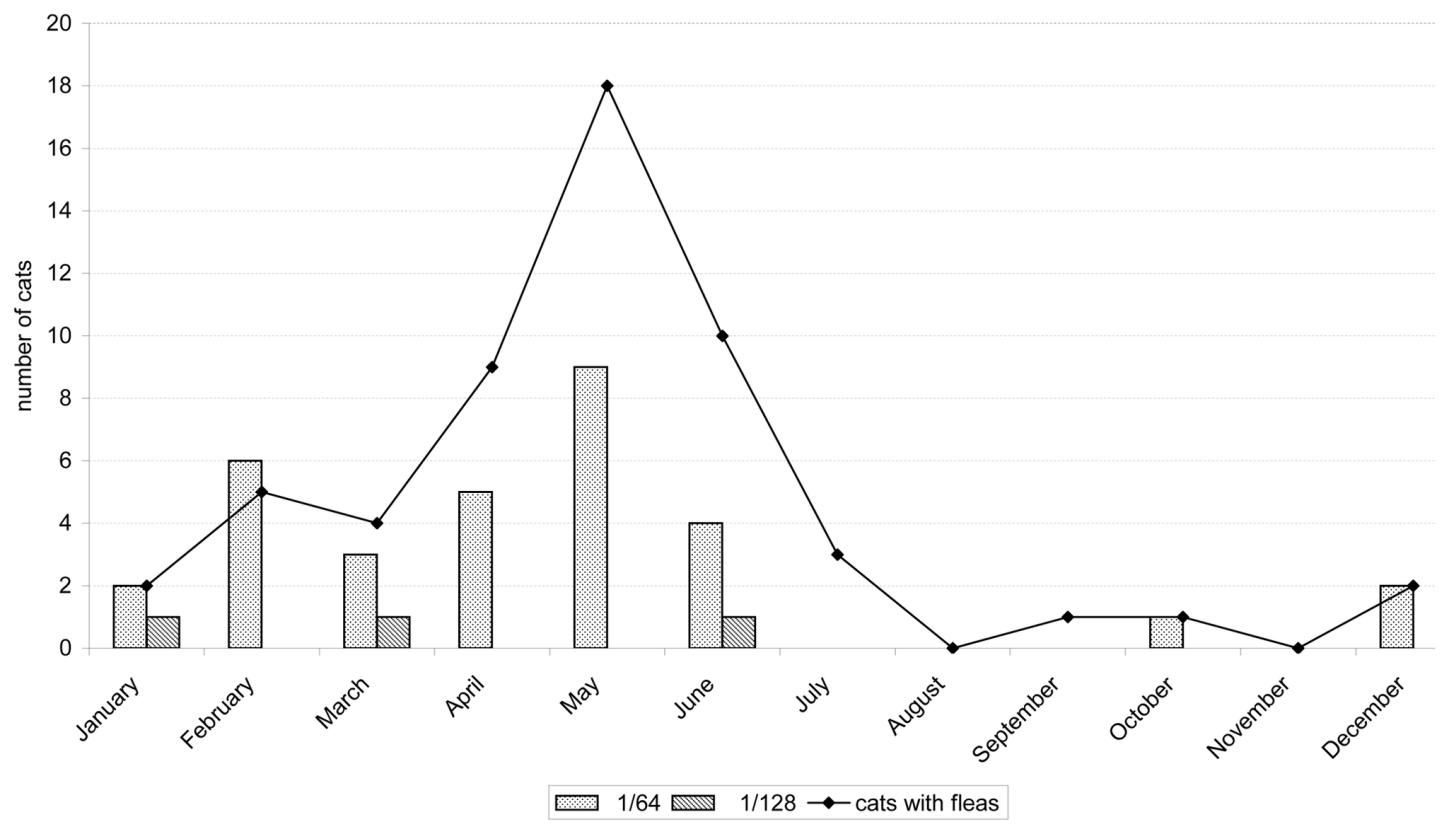
Annual distribution of infested animals and titres obtained by indirect immunofluorescence antibody assay.

Fleas were found on 55 (24.9%) cats. Geographical distribution of infested cats is shown in Figure 2. A statistically significant association was found between infestation and stray cats (p < 0.001), contact with animals (p < 0.001), and month of collection (p<0.001, Figure 3).

Twenty-nine (13.1%) cats showed some type of illness. The diseases observed were: abscess, dehydration, abortion, sepsis, cystitis, conjunctivitis, anemia, diabetes mellitus, diarrhea, fever, gingivitis, hepatic diseases, respiratory diseases, Feline immunodeficiency, poisoning, feline leukemia, breast lump, nasal lump, mouth infection, urinary tract infection, worms, and uterus infection. Fifty-five percent of stray cats (p < 0.05), and 55.6% (p=0.001) of infested cats were ill.

#### Sero-epidemiological study

**Figure 4:**
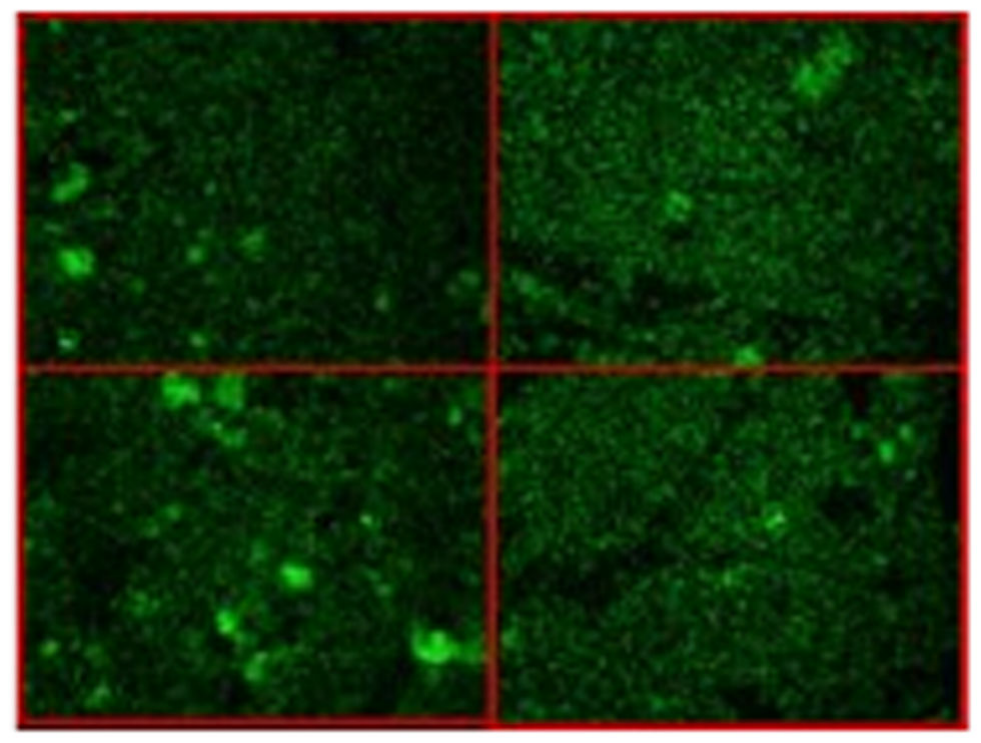
Images of positive indirect immunofluorescence assays.

Thirty-five (15.8%) sera had antibodies against *R* *. typhi* (1/64: 32 [14.5%], 1/128: 3 [1.4%]) (Figure 4). Distribution of titres throughout the year is shown in Figure 3. Cats with titres of 1/128 were healthy adults, from urban area near the coast, and they were not infested. Two of these lived in apartments, and the other one lived in a house.

The mean age of seropositive cats was 3.44 ± 3.09 years (0.5-11 years) and the mean age of seronegative cats was 3.98 ± 4.37 years (0.4-17 years) (non-significant). The relationship between the seropositivity and the surveyed items is shown in Table 1. There was no statistically significant association between any of the items and seropositivity or titres.

Outdoors activities were reported in 43 pets. A new variable called outdoors/indoors was built. Stray cats and pets with outdoors activities were considered as “Outdoors cats”. Fifteen cats with outdoors exposure were seropositive (non- significant).

Eleven seropositive cats had fleas (50% stray cats, 30.8% adults). The geographical distribution of seropositive cats is shown in Figure 2.

#### Molecular detection

Whole-blood sample in EDTA vacutainers was collected in 23 seropositive cats. In addition of these, samples from 23 seronegative cats were analysed. Five seropositive cats (numbers: 32, 39, 44, 170, 281) were positive by *R* *. typhi* -specific PCR assay (*omp*B). The sequences were identical among them and with those of *R. typhi* in the GenBank (CP003398, CP003397, AE017197, L04661). Table 2 shows information concerning these cats. Cat number 32 was also positive by PCR targeting *gltA* gene. However, the amount of DNA amplified was not enough to analyse it by sequencing. No amplification was obtained in seronegative cats.

**Table 2:** Demographic information from cats with positive results by molecular detection.

**Sample**	**32**	**39**	**44**	**170**	**281**
**Month of collection**	March	April	April	March	December
**Sex**	Female	Female	Male	Male	Male
**Age**	2 years	2 years	6 months	> 1 year	8 years
**Living place**	Apartment	Apartment	Apartment	Street (Stray cat)	Apartment
**Demographic area**	Urban	Urban	Urban	Urban	Urban
**Ectoparasites**	No	No	No	Fleas	No
**Illness**	No	No	No	Respiratory disease, feline immunodeficiency	No
**Contact with animals**	Cats	Dogs	No	Cats	No
**Titres**	1/128	1/64	1/64	1/64	1/64
**Real-time PCR (blood):**					
***omp*B**	+	+	+	+	+
***glt*A**	+	neg^1^	Neg	Neg	neg
**Real-time PCR (culture):**		NP^2^	NP	NP	
***omp*B**	+				neg

^1^ neg: negative

^2^ NP: not performed

#### Culture

Cultures were carried out in those cats in which rickettsial DNA was detected, and whose whole-blood samples were collected in heparin vacutainers (numbers: 32, 281). On day 40, a few rickettsiae were observed in Gimenez staining of cat 32. Fluorescence was observed inside few Vero cells of cat 32 incubated with antibodies against *R* *. typhi* . Presence of rickettsial DNA was detected in culture from cat 32. The sequence obtained was identical to those of *R* *. typhi* in the GenBank (CP003398, CP003397, AE017197, L04661). However, culture could not be established subsequently.

### Fleas’ study

All fleas were *Ctenocephalides felis. omp*B *R* *. typhi* -specific PCR yielded positive results in 44 (55%) fleas: 33/64 [51.6%] of fleas from kennels, 11/16 [68.8%] of those from Veterinary Clinics (non-significant). Nine amplicons were chosen at random and sequenced. The sequences were identical among them and to those of *R. typhi* in the GenBank (CP003398, CP003397, AE017197, L04661).

Presence of *Rickettsia* *felis* had been studied in 78 of the same fleas (previously published [21]). Twenty (25.6%) of these fleas contained DNA of both species (10/63 [15.8%] of fleas from kennels, and 10/55 [66.7%] of those from veterinary clinics)*.*

## Discussion

*Rickettsia* *typhi* is the aetiological agent of murine typhus. This disease can be misdiagnosed or underreported due to its non-specific symptoms, or the absence of epidemiological criteria [2–5,12]. Seroepidemiological studies have demonstrated the presence of *R* *. typhi* infection in human populations from many Spanish regions [12–15]. However, while Southern Spain is considered as an endemic area [5,7,8], few clinical cases have been reported in other regions, most of them in the Canary Islands [9–11]. Interestingly, some areas with no or few clinical cases show higher prevalences than those found in Southern or Canary Islands. Therefore, murine typhus may be underreported. Catalonia is a Mediterranean region in Northeastern Spain. Many cases of murine typhus have been described in the Mediterranean area, which is considered a risk area [22]. Previous results showed a considerable proportion of people from our region with past *R* *. typhi* infection [12]. Thus, it was necessary to identify reservoirs and vectors involved in *R* *. typhi* cycle and in close contact with people. Even though a peridomestic cycle involving cats and their fleas has been described [1], there is not much information, especially focused on the natural infection in cats. In this study, the presence of *R* *. typhi* in cats and fleas is demonstrated.

Although human *R* *. typhi* infection directly from cats has not been described, association between high rates of infected cats has been related to human cases of murine typhus. For instance, 90% of seropositive cats were found in an area of Los Angeles where human cases were described, whereas all cats living in a control area, without human clinical cases, were seronegative [16]. Recently, an outbreak of murine typhus in Austin was described [4]. Cats, dogs and opossums analyzed during this outbreak were seropositive whereas raccoons and rats were negative. Cats cannot only act as transport host of infective fleas into the human environment [16], but also they are susceptible to infection, and produce antibodies against it [16,17]. In fact, 15.8% of cats studied in our region were seroreactive. Moreover, a direct detection of *R* *. typhi* in some of these cats was obtained by PCR. Even though isolation of *R* *. typhi* was not achieved, results obtained by culturing also seem to point to the presence of *R* *. typhi* infection in the cats. To our knowledge, this is the first time of the *R* *. typhi* detection in naturally infected cats. As it was suspected, the rate of molecular detection was lower than seroprevalence. *R* *. typhi* is an intracellular pathogen and it could be found in tissues, as a consequence, bacteraemia may be short or not detected with only one sample. Moreover, as titres found in our study are not very high, the higher seroprevalence could also be due to a past infection with persistent antibodies.

It is important to highlight that many seropositive cats, even cats in which *R* *. typhi* has been directly detected, lived in close contact with humans. Like other studies [23,24], although stray cats or cats with outdoor activities may be more exposed to *R* *. typhi* infection, there were no significant differences among seropositivity and either habitat or outdoor activities.

There was no association between seroprevalence and illness. This fits in with the fact that cats may present subclinical infection [16,17]. Besides, *R* *. typhi* was detected by PCR in four healthy cats. One of these was the cat no. 32 which also had the highest titres, and a positive culture.

Cats are often infested by fleas. In fact, almost half the seropositive cats were infested, and half of these were pets. Taking into account that more than half the fleas surveyed in our study presented *R* *. typhi* DNA, vector control is very important, even in pet cats. On the other hand, four of five cats in which molecular detection was positive, lived in apartments and did not have ectoparasites. One of these cats presented positive results in culture too. In this case, its infection could be recent. Consequently, if infected cats are in close contact to humans, they may not present clinical symptoms, and they may not be infested, more controls on pets are necessary.

It has been suggested that kittens have lower rates of seropositivity due to their shorter contact with the microorganism. However, seroprevalence observed in kittens were similar to that observed in adult cats. Other studies have described the same results [23].

It has been described that murine typhus tends to be seasonal. Although cases can be detected throughout the year, the highest prevalences are found in summer, whereas a few cases are described in winter [7,10,25]. These results are in accordance with the seasonality of infestation, which may tend to be higher during summer. According to this, the highest percentage of our infested cats was found between April and June. However, 44 of the 68 stray cats (with higher proportion of infestation) were surveyed in these months, and, thus, association between infestation and seasonality is not as accurately as it should be. On the other hand, although 19 seropositive cats were found between March and June; there were 11 seroreactive cats in winter and *R* *. typhi* was detected by PCR in one of these. Therefore, despite seroreactive cats in winter mainly reflect the persistence of antibodies over time, some cats may also be infected in this season.

The present study is the first molecular detection of *R*. *typhi* in *Ctenocephalides felis* from Spain. There are not many studies on the presence of *R* *. typhi* in *C. felis* [25–27]. Our elevated prevalence suggests that *C. felis* may play a role in the epidemiological cycle of *R* *. typhi* . Considering *C. felis* avidly feeds on humans, it could transmit *R* *. typhi* to them. *C. felis* has a broad host range and is frequent on pets. It should be pointed out that fleas analysed not only were collected in kennels but they were also collected on cats attended at veterinary clinics, and therefore, living in a household.

Eleven (13.8%) fleas contained DNA from both *R* *. felis* and *R* *. typhi* . Although there may be an interspecific competition between rickettsiae in arthropods [26], Noden demonstrated that cat fleas could be infected with *R* *. felis* and *R* *. typhi* , and could maintain both species [28]. On the other hand, other authors suggested that fleas containing *R* *. felis* may not acquire *R* *. typhi* as readily as uninfected fleas. Although our data could not analyse this fact, dual infection was demonstrated as it was observed in *X* *. cheopis* [29].

## Conclusions

Evidences of presence of *R* *. typhi* in cats, and fleas have been shown in this study. Percentages observed were higher than those observed in the human population because animals usually present more exposure to vectors. Risk of infection in animals with close contact with humans is similar to that observed in stray animals or those living in kennels. Absence of ectoparasites or clinical symptoms does not guarantee that the animal is not infected. Although infection may be a seasonal, cases may appear throughout the year. Therefore, probably cases of murine typhus may be misdiagnosed in our area considering infected animals could be in close contact with people, past human infection has been detected in our region [12], and one clinical case was recently described near our area [30]. This is of great importance because delayed diagnosis and inappropriate therapy are related to severity and death.
